# Changing Food Consumption and Nutrition Intake in Kazakhstan

**DOI:** 10.3390/nu14020326

**Published:** 2022-01-13

**Authors:** Mengmeng Jia, Lin Zhen, Yu Xiao

**Affiliations:** 1 Institute of Geographic Sciences and Natural Resources Research, Chinese Academy of Sciences, A11 Datun Road, Chaoyang District, Beijing 100101, China; jiamm.19b@igsnrr.ac.cn (M.J.); xiaoy@igsnrr.ac.cn (Y.X.); 2 College of Resources and Environment, University of the Chinese Academy of Sciences, 19A Yuquan Road, No. 19, Shijingshan District, Beijing 100049, China

**Keywords:** food consumption, nutrition, influencing factors, Kazakhstan

## Abstract

Food resource is an important bond that connects human beings and nature. In this study, we investigated the changes in food consumption and nutrition intake in Kazakhstan from a spatial and temporal perspective, from 2001 to 2018. The data were obtained from the Bureau of Statistics, international organizations and our social interview work. After the start of the 21st century, it was found that per capita food consumption significantly increased; however, the consumption of crop, vegetables and milk decreased. Per capita meat consumption was similar in both urban and rural areas. However, some food consumption showed differences between urban and rural areas. Changes of food consumption quantity and structure also had some effects on nutrient intake and the proportion of nutrients. Per capita energy intake in the national, urban and rural areas all increased remarkably. The energy intake changes in eastern states increased much more than that in western states. Protein intake in rural and urban areas was similar; however, the gap between carbohydrates and fat intake in urban and rural areas increased. The intake of protein, carbohydrates and fat in different states showed the same trend. Food consumption and nutrition intake are affected by economic, social and ecological factors.

## 1. Introduction

Food consumption and security have always had a significant relationship with social stability and national sustainable development. The impact of the COVID-19 pandemic around the world has caused more serious challenges to food consumption patterns and nutrition situations [1,2]. The current status of food in the world has intensified the food crisis and attracted more attention from worldwide researchers to search for more sustainable food changes.

Studies on food security have mostly focused on factors that affects food security including climate change [3], international food prices and stable food production [4], the relationship between crop yield and livestock feed and greenhouse gas emissions from agriculture and land use changes [5,6,7], maintenance of food security from both the demand and supply sides [8], strategies for sustainable resource management [9]; additionally, studies have focused on types of food such as aquaculture [10] or a specific staple food such as wheat [11]. Although there have been some studies on food nutrition [12,13,14], many studies have focused on specific groups of people such as children [12] and specific types of foods such as beef [15], and there still have been few studies on total food consumption and its nutrients from several aspects and levels. At a national level, food security is generally assessed based on the average energy intake per capita in relation to the actual needs which are determined in accordance with minimum recommended nutritional standards.

Currently, food security issues are often considered on a nation-wide basis, within country groups [9,16,17,18] or at a worldwide level [19,20,21,22]. Studies on nutrition in Kazakhstan have focused on children or older people [12,23,24,25,26], with limited studies on the nutritional status of the total population. Moreover, there is more research on specific foods such as livestock [27], fruits and vegetables [28], but there are limited studies focusing on the total food consumption and its nutrition. As to theoretical methods, it is observed thanks to group interviews or questionnaires [29], or analysis models about climate shocks on specific food nutrition in Kazakhstan [30], that current research is still lacking regarding the analysis of different food consumption habits and nutritional changes at the national and state levels. In addition, the influencing factors affecting Kazakh food consumption have been another main research area, but the focus has been on a few macro factors such as food production quantity and imports [27,31], with a lack of factors relating to consumers such as age and household size.

Food consumption and its nutritional changes play a vital role on reflecting the living situation of a country or area. The purpose of this study is to explore the trend characteristics of Kazakh food consumption and the status of food nutrition at the national urban-rural levels and to identify the current status and changes in food nutrition from different spatial and temporal aspects at the national and state level.

## 2. Data and Method

### 2.1. Data Resources

In this study, total food consumption included plant-based and animal-based food; plant-based food mainly consisted of crop, vegetables, fruit and sugar; and animal-based food consisted of meat, milk, oil and cream, fish and eggs. The food consumption data were obtained from the Republic of Kazakhstan Bureau of National Statistics (https://stat.gov.kz/, accessed on 15 December 2020). The population data were from the World Bank (2018); the influencing factors such as GDP, import and cultural customs were obtained from the Food and Agriculture Organization of the United Nations (FAO, http://www.fao.org/faostat/en/#data, accessed on 15 December 2020) and team surveys. The transformation index was obtained from the food macronutrient conversion rate of food items in Russia (https://www.rlsnet.ru, accessed on 13 November 2020). The states in this analysis, from 2001 to 2018, referred to 16 states that consisted of 2 municipalities and 14 states.

### 2.2. Method

Research on food consumption depended on some important equations. Food consumption for energy and nutrients such as fats, protein and carbohydrates were achieved by introducing conversion factors and specific equations and explanations, as shown below:

(1) $$C_{r}=C_{i}/C_{t}\times 100\%=C_{i}/\left(C_{p}+C_{a}\right)\times 100\%$$

where C was the consumption quantity, *i* was the specific food item (*i* = 1–10), *t* was the total quantity, *p* was plant-based food, *a* was animal-based food, *r* was the percentage of the total. Therefore, $C_{i}$ was the specific food consumption quantity, $C_{p}$ was the consumption quantity of plant-based food, $C_{a}$ was the consumption quantity of animal-based food, and $C_{r}$ was the specific food consumption percentage of total food consumption.

(2) $$C_{F}=C_{\bar{U}}\times P_{U}+C_{\bar{R}}\times P_{R}$$

where $C_{N}$ was the total food consumption of national residents, $C_{U}$ was the total food consumption of all urban residents, $C_{R}$ was the total food consumption of all rural residents, $C_{\bar{U}}$ was the per capita food consumption of urban residents, $C_{\bar{R}}$ was the per capita food consumption of rural residents, $P_{U}$ was the population in urban area and $P_{R}$ was the population in rural areas.

(3) $$N_{i}=C_{\bar{i}}\times F_{i}$$

where $N_{i}$ was the nutrient content of daily food consumption, $C_{\bar{i}}$ was the per capita food consumption every day, F was the conversion factor from food consumption to associated nutrient content, $F_{i}$ was the conversion factor of food item *i* (*i* = 1–10). The values of the conversion factors were from https://www.rlsnet.ru (accessed on 13 November 2020) (Table 1).

## 3. Results Analysis

### 3.1. National, Urban and Rural Per Capita Food Consumption

**Figure 1:**
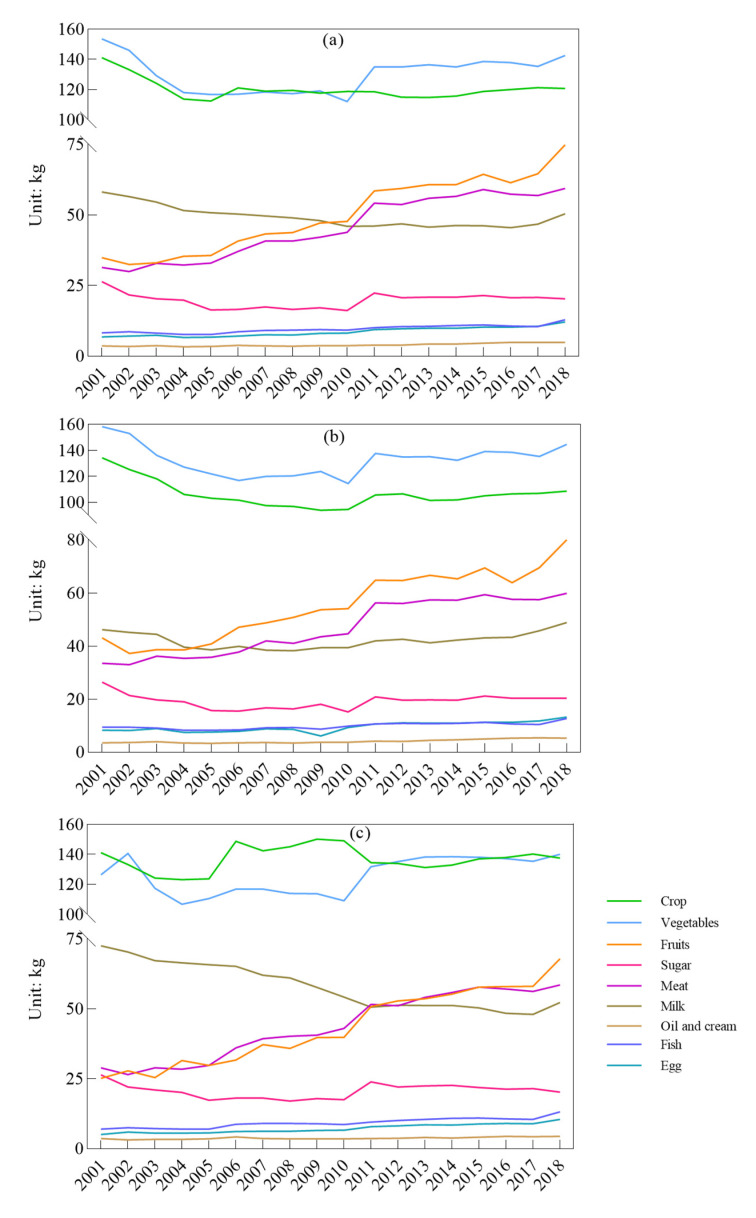
Per capita annual food consumption: (**a**) national; (**b**) urban; (**c**) rural areas.

In this section, we analyze and compare the changes in national, urban and rural per capita food consumption data, from 2001 to 2018 (Figure 1). The different per capita food consumption data indicated that the per capita consumption of crop and vegetables was higher than that of other types of food. The per capita consumption of crop in urban areas was much lower compared with that at the national level and in rural areas, while the per capita consumption of vegetables in urban areas was a little higher than that at the national level or in rural areas. During the study period, the per capita consumption of milk in rural areas showed a sharp decrease of 27.86%, while in urban areas it was relatively stable at a level of 42.14 kg per capita. The national per capita consumption of milk showed the same change trend as in urban areas, with a decrease of 13.40% over the study period. From 2001 to 2018, the per capita consumption of fruit and meat at the national level and in urban and rural areas increased, but the increases in fruit consumption were different, i.e., from 2001, at the national level, per capita consumption of fruit increased 115%, in urban areas it increased 85.61%, and in rural areas there was a 171% increase. The increase in per capita consumption of meat at the national level and in urban and rural areas was much less than that of fruit, i.e., 89.17%, 78.81% and 102.42%, respectively, during the study period. Per capita consumption of sugar showed a significant decrease. There were two changes in trend, in 2001 and 2011, when its consumption decreased slightly, and then remained stable. The national, urban, and rural per capita consumption of oil and cream remained stable at a low level, on average, of 3.97 kg, 4.16 kg and 3.74 kg, from 2001 to 2018. The increase in the national per capita consumption of both eggs and fish was stable and sustainable, with increases of 77.91% and 55.42% over the study period, respectively. However, the increase in per capita consumption of fish in rural areas was 4.46-fold, which was significantly more than the 2.23-fold increase in urban areas. The per capita consumption of fish at the national level, and in urban and rural areas, remained approximately stable at 9.59 kg during the study period.

**Figure 2:**
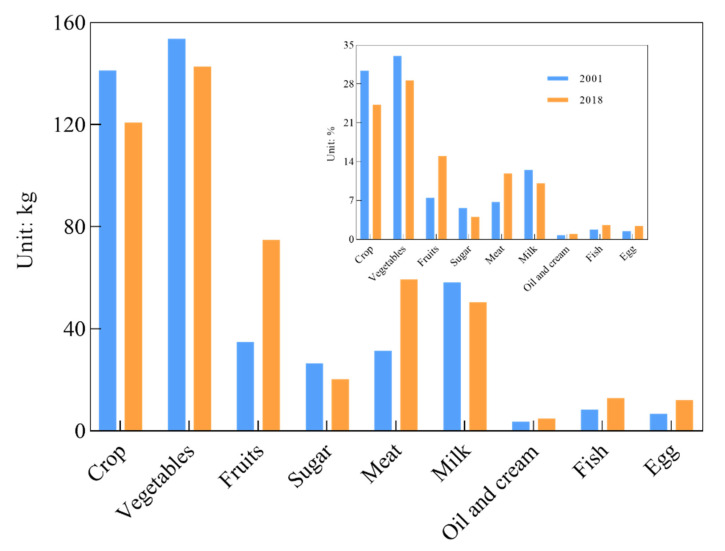
Food consumption structure and change.

Next, we describe the changes of per person annual food consumption in quantity and structure by different food resources through the study years. By comparing food consumption throughout the study period, significant increases in fruit and meat consumption were observed, with consumption levels of 15.03% and 11.92%, respectively, whereas oil and cream, fish and eggs increased somewhat (Figure 2). Some plant-based foods such as crop, sugar and vegetables, and animal-based foods such as milk, showed an opposite change trend compared to meat consumption; i.e., crop, sugar, vegetables and milk consumption decreased by 6.16%, 1.61%, 4.45% and 2.42%, respectively. This means that food consumption demand for fruit and meat increases with societal and economic development.

### 3.2. Changes of Nutrient Intake from Food Consumption

#### 3.2.1. Per Capita Nutrient Intake at the National Level

(1) Energy

**Figure 3:**
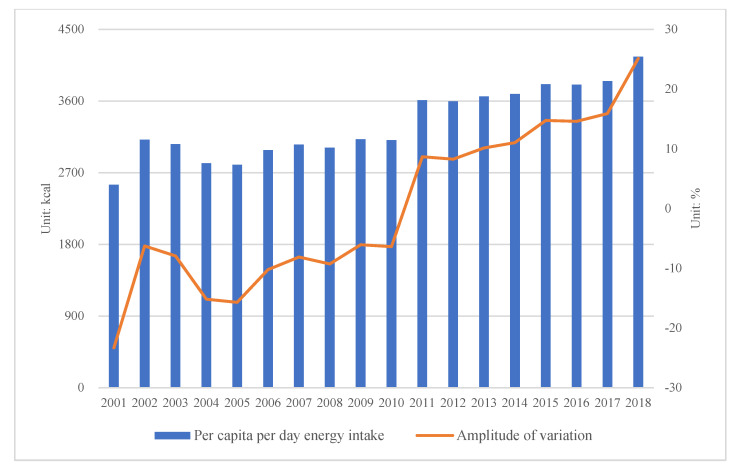
Change of national per capita per day energy intake.

The consumption level of energy in Kazakhstan showed an increase trend from 2001 to 2018. As shown in the figure below, per capita energy intake was 2547.3 kcal/day in the first year of the study (Figure 3). In 2002, the energy intake significantly increased by 22.22% compared with that of the year before. Although, energy intake increased in 2003, the per capita food consumption decreased to 3057.97 kcal/day which was 1.78% less than that of the year before. From 2004 to 2010, the per capita energy intake mainly remained at an average level of 2984.87 kcal/day, with a fluctuating change trend. From 2011 to 2017, per capita food consumption mainly remained at 3716.93 kcal/day, and the change trend in this period was similar to that of the period from 2004 to 2010; i.e., both change trends were unstable and fluctuated slightly. The change trends of the two periods show similarities, but the average food consumption for dietary energy from 2011 to 2017 was 24.53% more than that in the previous period, i.e., from 2004 to 2019. In 2018, per capita food consumption for energy intake reached the new level of 4156.67 kcal/day, which was the first time that the per capita food consumption exceeded 4000 kcal/day. According to the EAT-Lancet Commission (EAT), research results suggested a per capita energy intake of 2500 kcal/day [32]; therefore, it was obvious that the Kazakh daily food consumption was much more than the EAT recommended level.

**Figure 4:**
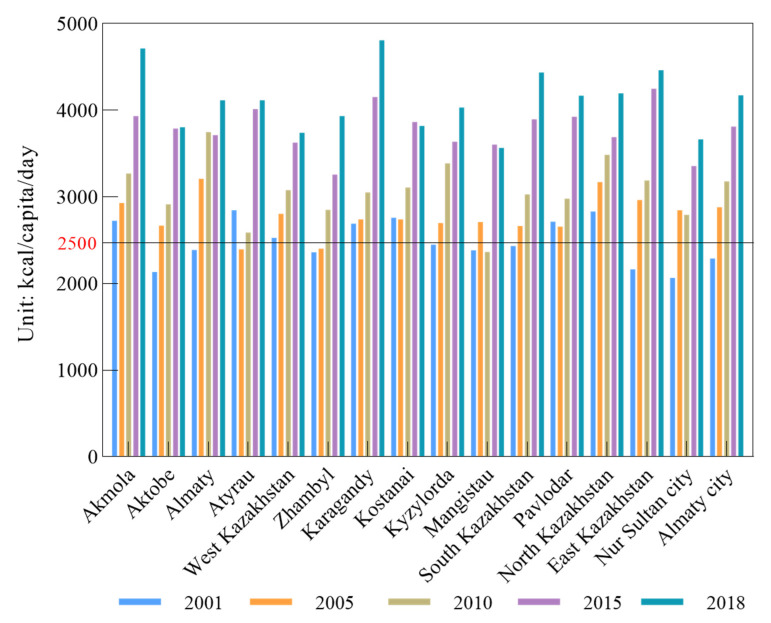
Changes in per capita per day energy intake in different states and municipalities.

The spatial change in energy intake showed significant diversity. In the first year of the study period, the energy intakes in central states were much higher than those in other states located in other regions (Figure 4). However, just four years later, the spatial layout of energy intake changed significantly, in that the core energy intake area had moved from the central to the southern states such as Almaty, South Kazakhstan and Almaty city. The highest energy intakes were in the southern states, Almaty and Almaty city. The energy intakes in these two states increased by 16.68% and 10.30% compared with those in the same states in 2010. At the same time, there was one area’s energy intake that decreased, which was Mangistau. In 2015, the energy intake in all states improved to different extents compared with that of five years ago. A comparison of energy intake in each year showed that the largest increase in energy intake was 55.08% in 2015, which appeared in Atyrau. According to the analysis, it was found that just one area’s energy intake experienced a slight decrease of 0.87% in 2015, which was Almaty. In 2018, compared to three years ago, it was observed that in the western states, the energy intake did not significantly change and remained stable. The energy intake in central and eastern states significantly increased. Interestingly, the largest increase in energy intake in 2018 was 20.81%, which was in Zhambyl. The new energy intake core states had moved to the central states which were Karaganda and Akmola.

Compared to the EAT’s recommended energy intake of 2500 kcal/capita/day, until 2015, the energy intake of all states and municipalities all exceeded the recommended level. In particular, in 2018, the energy intake in the central state of Karagandy was 4806.97 kcal/capita/day, which was approximately 1-fold more that of the recommended level. There were still some states’ energy intake levels below the recommended level, until 2010, and the difference between the state energy intake levels and the recommended intake level gradually became larger.

(2) Protein, fat, and carbohydrates

**Figure 5:**
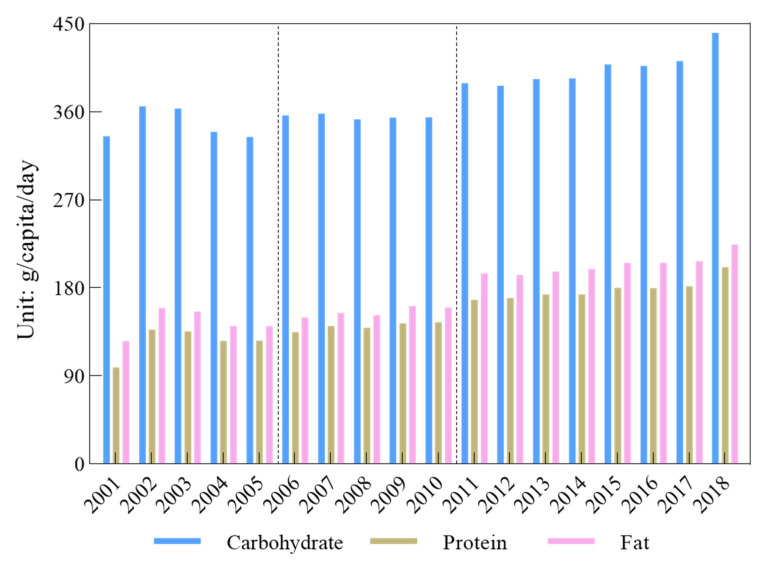
National per capita per day intakes of carbohydrates, protein and fat.

It was significant that the change in the national per capita per day intake of carbohydrates, protein and fat showed periodic change trends, with an overall increase of 31.55%, 103.87% and 78.37%, respectively (Figure 5). It could be divided into three stages according to the change trend characteristics. In the first stage, from 2001 to 2005, the change trend of both carbohydrates and fat intake showed an increase first, and then a decrease, while the change trend of protein intake increased and decreased repeatedly. In the second stage, from 2006 to 2010, the overall change trend was rather small in that fat intake increased and decreased repeatedly, while the change trend for the intake of both carbohydrates and protein increased firstly, decreased secondly and then increased again slightly. In the stage from 2011 to 2018, the overall increase change trend was remarkable, i.e., the increases in the national per capita per day intake of carbohydrates, protein, and fat were 13.25%, 19.88% and 15.16%, respectively.

**Figure 6:**
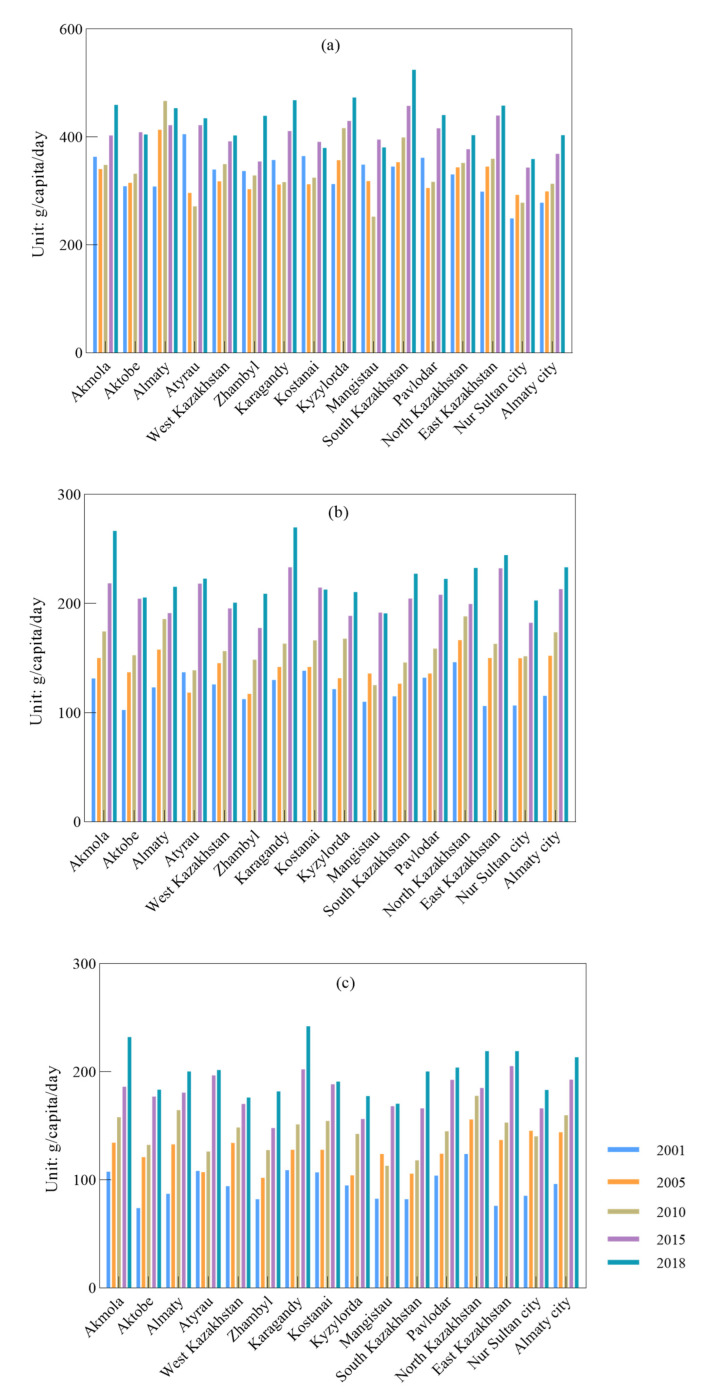
Spatial change of per capita per day nutrient intake: (**a**) carbohydrates; (**b**) protein; (**c**) fat.

Per capita per day intake of carbohydrates in all the states and municipalities showed various change trends, including a continuous increase, first a decrease and then an increase, and repeated increasing and decreasing (Figure 6a). It was remarkable that the highest protein intake was in South Kazakhstan with 524.83 g/capita/day. South Kazakhstan, Kyzylorda, North Kazakhstan, East Kazakhstan and Almaty city showed a continuous increase in protein intake of 51.31%, 22.00%, 53.41% and 44.98%. These five areas were mainly located in the east of Kazakhstan. The total per capita per day protein intake in most of the states and municipalities showed continuous increases, except for Atyrau, Mangistau and Nur Sultan city, where significant fluctuations in protein intake occurred in different years; i.e., protein intake in Atyrau decreased in 2005, whereas in both Mangistau and Nur Sultan city, it decreased in 2010 (Figure 6b). The two areas with the highest protein intake were in the central Kazakhstan states of Karagandy and Akmola with 242.16 and 232.24 g/capita/day, respectively. According to the comparisons, it was observed that the largest increase of 188.40% was in East Kazakhstan. During the entire study period, per capita per day fat intake of each state and municipality showed the same change trend as protein, i.e., an increase, although to a different extent (Figure 6c). It was significant that the highest fat intakes were in Karagandy and Akmola with increases of 107.67% and 103.13%, respectively. This might be due to the local high yield level and crop supply. Fat intake in all the states and municipalities showed a continuous increase, except for Atyrau, in which the fat intake trend was first a decrease, and then an increase.

#### 3.2.2. Per Capita Nutrient Intake in Urban and Rural Areas

(1) Energy

**Figure 7:**
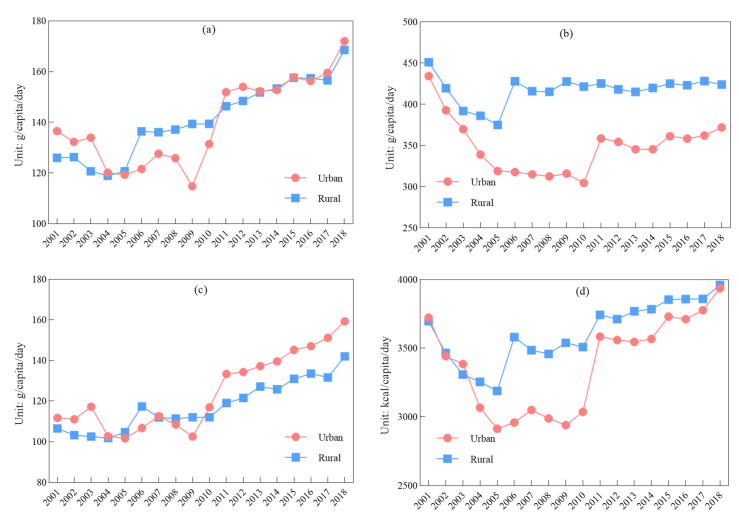
Changes in urban and rural per capita everyday nutrient intake: (**a**) protein; (**b**) carbohydrates; (**c**) fat; (**d**) energy.

According to the comparisons and analyses of per capita energy intakes in urban and rural areas, although the energy intakes in both areas increased in the final year compared with those in the first year of the study, there were significant fluctuations over time (Figure 7d). The change in daily energy intake in rural areas was much larger than that in urban areas, especially from 2003 to 2011. Per capita daily energy intake in rural areas was higher than that in urban areas, and the greatest difference between them was 621.94 kcal/day which was in 2006. Since then, the difference was much smaller and the energy intake in urban and rural areas was rather similar in 2018. According to the EAT, the recommended healthy per capita energy intake was 2500 kcal/day. The energy intakes in both urban and rural areas were above the recommended level, and the difference between the recommended and actual energy intake had become much greater since 2011.

(2) Protein, fat and carbohydrates

During the first half of the study period, the per capita per day intakes of protein, carbohydrates and fat, showed more significant fluctuations than those in the latter half of the study period (Figure 7a–c). Protein intake in urban areas was lower than that in rural areas, but several years later, the situation was reversed. Then, protein in both urban and rural areas increased and tended to be equal. Although, fat intakes in rural and urban areas in the early years of the study had similar change trends as that of protein uptake, since 2010, fat intake in urban areas had exceeded that in rural areas, and the difference between the areas was widening compared to those in the first year. The intakes of carbohydrates in rural areas were always higher than those in urban areas; the difference in the intakes of carbohydrates between rural and urban areas was widening, with the largest difference of 38% in 2010. Although the difference became somewhat narrower, it still remained at 14% in 2018.

## 4. Discussion

The total per capita food consumption, by residents who lived in both urban and rural areas, showed a significant increase in quantity. The percentage of fat intake of the total energy intake significantly increased at the national, state and municipal levels, while the share of carbohydrate intake showed some decrease and that of protein intake remained relatively stable. Compared with median energy intake of 2085 kcal in Australia in 2012 [33], the national energy intake level in Kazakhstan was much higher. More energy intake than energy expenditure easily causes energy imbalance [34], and this is one of the affecting factors of chronic diseases [35]. Moreover, excessive fat intake is associated with bodyweight and diseases such as cardiovascular disease, especially in developing countries [35]. However, some researchers have studied the Korean adult population and found that frequent consumption of dairy, including milk, might have a beneficial effect on periodontal diseases [36]. More attention should be given to fat intake and applications for healthy living and scientific food consumption. With regard to the factors influencing food consumption and changes in nutrients, discussions mainly focus on cultural customs, GDP, as well as import and production changes affecting some important foods.

Culture has a significant effect on residents’ life, attitudes, behaviors and values which influence what we do and how we do it [37,38,39]. We usually chose edible food quantity and quality based on the culture we have experienced [40]. The cultivation of food consumption structure and habitat have complicated the relationship with local cultures and customs [41]. Asian countries mostly consume staple foods from cereals, while in European countries, residents prefer meat such as beef and mutton. For example, one study found that in Bangladesh there is still a notion that “rice or meat was more important than vegetables”, which suggests that food culture is a more prevalent factor than mere affordability [42,43]. The people who traditionally live in Kazakhstan are mostly nomadic [44]. Their food mainly consists of meat, eggs and milk [27], which was highlighted by the per capita food consumption in both urban and rural populations, i.e., that per capita meat consumption stably increased by 78.81% in urban areas and 102.42% in rural areas during the study period. Compared with other countries, meat food consumption in Kazakhstan was much lower than that in Russia (67.79 kg, in 2008), but was much higher than that in China (28.29 kg, in 2009) [45]. These three countries had similar gradually increasing meat consumption trends.

One important reason to expand food imports from other countries is to mitigate domestic food consumption and the ecological pressure that is caused by an increase in population and the cost of production, or limited available land use [46,47]. It is well known that the production of fruit and vegetables is not adequate for domestic consumption in Kazakhstan; however, the per capita consumption of both vegetables and fruit has changed, i.e., the per capita consumption of vegetables fluctuated, and the per capita consumption of fruit increased by 1.15-fold in the last year, compared with that in the first year, of the study. This was mostly due to international imports that helped to meet the gradual increase in fruit and vegetable consumption. The quantity of imported fruit and vegetables increased by 22.67-fold and 60.31-fold, respectively, in the last year compared with those in the first year. Although food imports could deal with many countries’ domestic food crises, food imports are also more dependent on other foreign countries, and risks from anti-globalism around the world and the worldwide pandemic have aggravated the potential international risk [48].

Food consumption is associated with the GDP which is an important foundation for household food purchasing [49,50]. The effect of the GDP on food consumption is significant, and therefore it plays a noteworthy role in improving residents’ food consumption quantity and structure, especially in rural areas [49,51]. The GDP in Kazakhstan showed a significant growth, from 7.59 × 10^10^ USD dollars to 2.04 × 10^11^ USD dollars, which increased by 1.69-fold during the study period of eighteen years. Sustainable growth of the GDP resulted in a strong foundation for purchasing more and more diversified food. Growth in the GDP usually meant that households’ disposable incomes had increased to some extent, which could directly improve the quality of life of residents [52]. According to the change in final consumption expenses of households over the study period, it was found that this expense also significantly increased with the same trend as the GDP [53].

The level of food production, especially the staple food such as rice and wheat, particularly plays an important role in local food consumption and nutrition in developing countries [54,55], especially in low-income countries [56]. Because the people who live in non-rich countries or areas cannot obtain a great deal of imported food, they depend on local food production [57,58]. In order to support an increasing population, it is urgent to improve suitable local food production [59]. Although Kazakhstan has been increasing the quantity of imported fruit and vegetables, at the same time, it has also insisted on improved crop production and animal breeding quantity. The production of wheat, rice and potato has experienced different increases which have provided a strong foundation for domestic food consumption and other needs such as feed for animals. The production of three types of crops, i.e., wheat, rice and potato increased 4.05%, 65.74% and 48.95%, respectively, compared with those in the first year of the study. The people of Kazakhstan belong to a nomadic ethnic group; therefore, keeping a relatively stable breeding quantity of animals is important to meet domestic consumption needs. The breeding quantities of cattle, sheep and chickens increased over the eighteen-year study period, i.e., an increase of 74.13%, 83.64% and 118.83%, respectively, compared with those in 2001. Core crop production and animal breeding helped Kazakhstan to maintain a high independence in food consumption.

This study was carried out based mainly on statistical data. Although this type of data covered large spatial areas, this data could not illustrate the quantity of food wastage, and this may have an impact on reflecting the actual situation of food consumption and nutritional quantity. The results were not as compelling and targeted as dietary survey data to reflect the spatial changes in specific food consumption and nutritional situations in different states. The cultivation of food consumption structure and its nutrient situation included influencing factors such as the environment, economy and society, which needed more sustainable research for improvements. We plan to collect more information through a questionnaire [60] and interviews with key stakeholders [61] in future research.

## 5. Conclusions

According to the analyses and comparisons above, Kazakhstan experienced a series of changes in food consumption from 2001 to 2018. Important conclusions from our results include the following:

(1) Due to sustainable economic prosperity, people had more discretionary income to purchase food, and therefore, the total quantity of per capita food consumption significantly increased. However, at the national level, different kinds of food showed different change trends, such as the decreases in the per capita consumption of crop, vegetables and milk consumption, while other types of food consumption showed the opposite change trend. The per capita food consumption of meat, fish, fruit, eggs, oil and cream increased in both urban and rural areas, whereas crop and sugar consumption decreased. In addition, the per capita consumption of vegetables and milk in urban areas showed opposite change trends compared with those in rural areas. The changes in per capita consumption of both eggs and meat in rural areas were much greater that those in urban areas. Regarding food consumption structure, the proportion of meat in the per capita food consumption significantly increased at the national, urban and rural levels, and the proportion of crop per capita food consumption decreased somewhat.
(2) The change in food consumption quantity and structure had some effect on nutrient intake; the energy intake also achieved a comparatively greater increase with an increase in food consumption quantity. Per capita energy intake at the national level, and in urban and rural areas, all significantly increased and exceeded the EAT recommended level of 2500 kcal/capita/day. The spatial change of energy intake in the eastern states increased more than that in the western states. The higher rate of increase in energy intake mainly focused in the eastern and northern states.
(3) The proportion of carbohydrates, protein and fat in urban and rural areas showed various changes. Protein intake in rural and urban areas was similar after repeated changes in several former years, but the differences in the intake of carbohydrate and fat in urban and rural areas became greater, i.e., carbohydrate intake in rural areas was higher than that in urban areas, and fat intake in rural areas was lower than that in urban areas. This was mainly because food consumption in rural areas included more crop, while food consumption in urban areas included more meat than in rural areas. The intake of carbohydrates, protein and fat in the different states showed the same change trend as the overall intake of these three nutrients, i.e., a significant increase, but specific states or municipalities during the study period showed some fluctuations.

Our study results reveal the situation and change trends in food consumption, nutrient intake as well as food consumption quantity and structure from spatial and temporal aspects. Nowadays, one of the most serious challenges we are facing is the COVID-19 pandemic, as it has a great impact on the economy and healthy living. It also inevitably affects food trade and the decline in transport efficiency, which also has an effect on domestic production to some extent because of the lack of external supplies. How the pandemic affects food consumption and nutrition in Kazakhstan will be one of the main focuses of research in the future. In order to improve food supply and consumption level, and deal with the negative impact of the pandemic, it is sensible to search for more scientific and sustainable ways and measures based on respecting actual food consumption and nutritional status. This will have a positive effect on the sustainable use and development of resources in Kazakhstan in the future.

## Figures and Tables

**Table 1:** Conversion factor of different food items per 100 g.

Number	Food Item	Energy (kcal)	Protein (g)	Fat (g)	Carbohydrates (g)
1	Crop	334	12	2.9	69.3
2	Meat	187	18.9	12.4	0.2
3	Fish	115	26	1.2	-
4	Milk	58	2.8	3.2	4.7
5	Eggs	157	12.7	11.5	0.7
6	Oil and cream	748	0.6	82.5	0.9
7	Fruit	46	0.4	-	11.3
8	Vegetables	28	1.8	-	5.4
9	Potato	83	2	0.1	19.7
10	Sugar	375	-	-	99.9

## Data Availability

Not applicable.

## References

[1] Boyaci-Gunduz C.P., Ibrahim S.A., Wei O.C., Galanakis C.M. (2021). Transformation of the food sector: Security and resilience during the COVID-19 pandemic. Foods.

[2] Chen K.Z., Mao R. (2020). Fire lines as fault lines: Increased trade barriers during the COVID-19 pandemic further shatter the global food system. Food Secur..

[3] Lake I.R., Hooper L., Abdelhamid A., Bentham G., Boxall A.B.A., Draper A., Fairweather-Tait S., Hulme M., Hunter P.R., Nichols G. (2012). Climate change and food security: Health impacts in developed countries. Environ. Health Perspect..

[4] Ghattas H., Barbour J.M., Nord M., Zurayk R., Sahyoun N.R. (2013). Household food security is associated with agricultural livelihoods and diet quality in a marginalized community of rural bedouins in Lebanon. J. Nutr..

[5] Valin H., Havlik P., Mosnier A., Herrero M., Schmid E., Obersteiner M. (2013). Agricultural productivity and greenhouse gas emissions: Trade-offs or synergies between mitigation and food security?. Environ. Res. Lett..

[6] Gerbens-Leenes W., Nonhebel S. (2005). Food and land use. The influence of consumption patterns on the use of agricultural resources. Appetite.

[7] Eberle U., Fels J. (2016). Environmental impacts of German food consumption and food losses. Int. J. Life Cycle Assess..

[8] Godfray H.C.J., Garnett T. (2014). Food security and sustainable intensification. Philos. Trans. R. Soc. B Biol. Sci..

[9] McLaughlin D., Kinzelbach W. (2015). Food security and sustainable resource management. Water Resour. Res..

[10] de Roos B., Roos N., Mamun A.A., Ahmed T., Sneddon A.A., Murray F., Grieve E., Little D.C. (2019). Linking agroecosystems producing farmed seafood with food security and health status to better address the nutritional challenges in Bangladesh. Public Health Nutr..

[11] Mwambo F.M., Furst C., Nyarko B.K., Borgemeister C., Martius C. (2020). Maize production and environmental costs: Resource evaluation and strategic land use planning for food security in northern Ghana by means of coupled emergy and data envelopment analysis. Land Use Policy.

[12] Turgambayeva A., Syzdykova A., Tulemisova A., Omarkulov B., Alihanova K. (2014). Analysis of state of early year children nutrition status in children’s home of Kazakhstan. FASEB J..

[13] Nolasco C.L., Soler L.S., Freitas M.W.D., Lahsen M., Ometto J. (2017). Scenarios of vegetable demand vs. production in Brazil: The links between nutritional security and small farming. Land.

[14] El Bilali H., Callenius C., Strassner C., Probst L. (2019). Food and nutrition security and sustainability transitions in food systems. Food Energy Secur..

[15] Mogensen L., Hermansen J.E., Trolle E. (2020). The climate and nutritional impact of beef in different dietary patterns in Denmark. Foods.

[16] Araujo-Enciso S.R., Fellmann T. (2020). Yield variability and harvest failures in Russia, Ukraine and Kazakhstan and their possible impact on food security in the Middle East and North Africa. J. Agric. Econ..

[17] Pyagay A., Zhekeyeva K., Aktailakova G., Iskakova M., Tulegenova Z. (2018). Ensuring food security of developing economy: Issues and perspectives. J. Agric. Sci. Technol..

[18] Skaf L., Buonocore E., Dumontet S., Capone R., Franzese P.P. (2019). Food security and sustainable agriculture in Lebanon: An environmental accounting framework. J. Clean. Prod..

[19] Beltran-Pena A., Rosa L., D’Odorico P. (2020). Global food self-sufficiency in the 21st century under sustainable intensification of agriculture. Environ. Res. Lett..

[20] Kuyper T.W., Struik P.C. (2014). Epilogue: Global food security, rhetoric, and the sustainable intensification debate. Curr. Opin. Environ. Sustain..

[21] Mekonnen M.M., Gerbens-Leenes W. (2020). The water footprint of global food production. Water.

[22] Tilman D., Balzer C., Hill J., Befort B.L. (2011). Global food demand and the sustainable intensification of agriculture. Proc. Natl. Acad. Sci. USA.

[23] Dangour A.D., Hill H.L., Ismail S.J. (2002). Height, weight and haemoglobin status of 6 to 59-month-old Kazakh children living in Kzyl-Orda region, Kazakhstan. Eur. J. Clin. Nutr..

[24] Hashizume M., Shimoda T., Sasaki S., Kunii O., Caypil W., Dauletbaev D., Chiba M. (2004). Anaemia in relation to low bioavailability of dietary iron among school-aged children in the Aral Sea region, Kazakhstan. Int. J. Food Sci. Nutr..

[25] Jensen S., Mazhitova Z., Zetterstrom R. (1997). Environmental pollution and child health in the Aral Sea region in Kazakhstan. Sci. Total Environ..

[26] Sharmanov T.S. (1990). Nutrition patterns of infants and their mothers in terms of infant-mortality in Kazakhstan. Vestn. Akad. Meditsinskikh Nauk. Sssr.

[27] Liang Y.H., Zhen L., Zhang C.S., Hu Y.F. (2020). Consumption of products of livestock resources in Kazakhstan: Characteristics and in fluencing factors. Environ. Dev..

[28] Goryakin Y., Rocco L., Suhrcke M., Roberts B., McKee M. (2015). Fruit and vegetable consumption in the former Soviet Union: The role of individual- and community-level factors. Public Health Nutr..

[29] Schwerin M., Schonfeld S., Drozdovitch V., Akimzhanov K., Aldyngurov D., Bouville A., Land C., Luckyanov N., Mabuchi K., Semenova Y. (2010). The utility of focus group interviews to capture dietary consumption data in the distant past: Dairy consumption in Kazakhstan villages 50 years ago. J. Dev. Orig. Health Dis..

[30] Tanaka T., Geyik O., Karapinar B. (2021). Short-Term Implications of Climate Shocks on Wheat-Based Nutrient Flows: A Global "Nutrition at Risk" Analysis through a Stochas-tic CGE Model. Foods.

[31] Kondybayeva S., Nurgazy S., Serik O., Mukhamediyev B., Sadykhanova G., Soliman K.S. (2018). Food market of Kazakhstan: Current state and innovative development directions. Innovation Management and Education Excellence through Vision 2020.

[32] (2019). Food in the Anthropocene: The EAT–Lancet Commission on Healthy Diets from Sustainable Food Systems. https://www.thelancet.com/pdfs/journals/lancet/PIIS0140-673631788-4.pdf?utm_campaign=tleat19&utm_source=HubPage.

[33] Waern R.V.R., Cumming R.G., Blyth F., Naganathan V., Allman-Farinelli M., Le Couteur D., Simpson S.J., Kendig H., Hirani V. (2015). Adequacy of nutritional intake among older men living in Sydney, Australia: Findings from the Concord Health and Ageing in Men Project (CHAMP). Br. J. Nutr..

[34] Bel-Serrat S., Ojeda-Rodriguez A., Heinen M.M., Buoncristiano M., Abdrakhmanova S., Duleva V., Sant’Angelo V.F., Fijalkowska A., Hejgaard T., Huidumac C. (2019). Clustering of Multiple Energy Balance-Related Behaviors in School Children and its Association with Overweight and Obesity—WHO European Childhood Obesity Surveillance Initiative (COSI 2015–2017). Nutrients.

[35] Popkin B.M., Adair L.S., Ng S.W. (2012). Global nutrition transition and the pandemic of obesity in developing countries. Nutr. Rev..

[36] Kyueun L., Jihye K. (2019). Dairy Food Consumption is Inversely Associated with the Prevalence of Periodontal Disease in Korean Adults. Nutrients.

[37] Walder P., Sinabell F., Unterlass F., Niedermayr A., Fulgeanu D., Kapfer M., Melcher M., Kantelhardt J. (2019). Exploring the relationship between farmers’ innovativeness and their values and aims. Sustainability.

[38] Smith J., Pearce B.D., Wolfe M.S. (2012). A European perspective for developing modern multifunctional agroforestry systems for sustainable intensification. Renew. Agric. Food Syst..

[39] Hawkesworth S., Dangour A.D., Johnston D., Lock K., Poole N., Rushton J., Uauy R., Waage J. (2010). Feeding the world healthily: The challenge of measuring the effects of agriculture on health. Philos. Trans. R. Soc. B Biol. Sci..

[40] Prescott J., Young O., O’Neill L., Yau N.J.N., Stevens R. (2002). Motives for food choice: A comparison of consumers from Japan, Taiwan, Malaysia and New Zealand. Food Qual. Prefer..

[41] Mak A.H.N., Lumbers M., Eves A., Chang R.C.Y. (2012). Factors influencing tourist food consumption. Int. J. Hosp. Manag..

[42] Mustafa S., Haque C.E., Baksi S. (2021). Low daily intake of fruits and vegetables in rural and urban Bangladesh: Influence of socioeconomic and demographic factors, social food beliefs and behavioural practices. Nutrients.

[43] Nam K.C., Jo C., Lee M. (2010). Meat products and consumption culture in the East. Meat Sci..

[44] Tiberghien G. (2020). Neo-nomadic Culture as a Territorial Brand for ‘Authentic’ Tourism Development in Kazakhstan. Eur. Asia Stud..

[45] Christine B., Lena K., Zhao Q., Ramona T., Thomas G. (2015). Economic growth and nutrition transition: An empirical analysis comparing demand elasticities for foods in China and Russia. J. Integr. Agric..

[46] Sadler M., Magnan N. (2011). Grain import dependency in the MENA region: Risk management options. Food Secur..

[47] Ali M., Marvuglia A., Geng Y., Robins D., Pan H.Y., Song X.Q., Yu Z.J., Sun H.P. (2019). Accounting emergy-based sustainability of crops production in India and Pakistan over first decade of the 21st century. J. Clean. Prod..

[48] Welburn J., Bier V., Hoerning S. (2016). Import security: Assessing the risks of imported food. Risk Anal..

[49] Mottaleb K.A., Rahut D.B., Kruseman G., Erenstein O. (2018). Evolving food consumption patterns of rural and urban households in developing countries A Bangladesh case. Br. Food J..

[50] Desiere S., Hung Y., Verbeke W., D’Haese M. (2018). Assessing current and future meat and fish consumption in Sub-Sahara Africa: Learnings from FAO Food Balance Sheets and LSMS household survey data. Glob. Food Secur.-Agric. Policy Econ. Environ..

[51] Guine R.P.F., Florenca S.G., Barroca M.J., Anjos O. (2020). The link between the consumer and the innovations in food product development. Foods.

[52] Aromolaran A.B. (2004). Household income, women’s income share and food calorie intake in South Western Nigeria. Food Policy.

[53] Landrigan T.J., Kerr D.A., Dhaliwal S.S., Pollard C.M. (2019). Protocol for the Development of a Food Stress Index to Identify Households Most at Risk of Food Insecurity in Western Australia. Int. J. Environ. Res. Public Health.

[54] Coelho F.C., Coelho E.M., Egerer M. (2018). Local food: Benefits and failings due to modern agriculture. Sci. Agric..

[55] Hlaing-Hlaing H., Pezdirc K., Tavener M., James E.L., Hure A. (2020). Diet quality indices used in Australian and New Zealand adults: A systematic review and critical appraisal. Nutrients.

[56] Leme A.C.B., Hou S., Fisberg R.M., Fisberg M., Haines J. (2021). Adherence to food-based dietary guidelines: A systemic review of high-income and low- and middle-income countries. Nutrients.

[57] Andriamparany J.N., Hanke H., Schlecht E. (2021). Food security and food quality among vanilla farmers in Madagascar: The role of contract farming and livestock keeping. Food Secur..

[58] Horst M., Gaolach B. (2015). The potential of local food systems in North America: A review of foodshed analyses. Renew. Agric. Food Syst..

[59] Bene C., Barange M., Subasinghe R., Pinstrup-Andersen P., Merino G., Hemre G.I., Williams M. (2015). Feeding 9 billion by 2050-putting fish back on the menu. Food Secur..

[60] Yang W.N., Zhen L., Wei Y.J. (2020). Food consumption and its local dependence: A case study in the Xilin Gol China. Environ. Dev..

[61] Cortner O., Garrett R.D., Valentim J.F., Ferreira J., Niles M.T., Reis J., Gil J. (2019). Perceptions of integrated crop-livestock systems for sustainable intensification in the Brazilian Amazon. Land Use Policy.

